# Complete Genome Sequence of *Sandaracinobacter* sp. Strain M6, Isolated from a Rocky Mountain in China

**DOI:** 10.1128/MRA.01415-20

**Published:** 2021-01-07

**Authors:** Ying Tang, Jun Huang, Cuiyang Zhang, Shiyu Bi, Zhaohui Guo, Qingshu Liu, Ping Lei

**Affiliations:** a Hunan Institute of Microbiology, Changsha, Hunan, China; University of Southern California

## Abstract

We report the complete genome sequence of *Sandaracinobacter* sp. strain M6 obtained by Oxford Nanopore and Illumina sequencing. The approximately 3.4-Mb genome sequence with a GC content of 67.65% provides essential data for future taxonomic studies and information for further investigating the metabolic characteristics of aerobic anoxygenic phototrophs.

## Abstract (precis)

We report the complete genome sequence of *Sandaracinobacter* sp. strain M6 obtained by Oxford Nanopore and Illumina sequencing. The approximately 3.4 Mb genome sequence with a GC content of 67.65% provides essential data for future taxonomic studies and information for further investigating the metabolic characteristics of aerobic anoxygenic phototrophs.

## ANNOUNCEMENT

Aerobic anoxygenic phototrophic bacteria (AAP) are a group of bacteriochlorophyll a-containing microbes. Currently, the AAP contains two marine genera and six freshwater genera, including *Sandaracinobacter* (1). Until now, the genus *Sandaracinobacter* has contained only two species with valid published names (2, 3).

The strain M6 was isolated from a soil sample collected on a rocky mountain in Changsha, Hunan Province, China (28.46°N, 113.18°E). The soil sample was surface spread onto SSE/HD agar (4). After 6 days, one yellow colony designated M6 was isolated and purified. The isolate was subcultivated routinely on rich organic (RO) medium at 30°C.

The 16S rRNA gene of strain M6 was first amplified by PCR using primers 27F and 1492R (5) and then ligated into a pMD-18T vector via TA cloning for subsequent sequencing. The closest phylogenetic neighbors of this sequence were identified using the EZBioCloud server (6), revealing the highest similarity (95.54%) to Sandaracinobacter sibiricus strain RB16-17, followed by Sandaracinobacter neustonicus strain PAMC 28131, with a similarity of 95.14%, both below the threshold of 98.7% for differentiating two species (7). Therefore, strain M6 was chosen for full-genome sequencing as supplemental information for the identification of a new species.

A single colony of M6 was inoculated into Erlenmeyer flasks containing rich organic (RO) medium and shaken aerobically at 28°C for 7 days (8, 9). The genomic DNA was extracted by a standard phenol-chloroform method and further purified by AMPure XP beads (Beckman Coulter, Brea, CA, USA) and then quantified and quality controlled using a Qubit 2.0 fluorometer (Thermo Fisher Scientific, Waltham, MA), Nanodrop software, and agarose gel electrophoresis (10). DNA was used for Oxford Nanopore and Illumina sequencing. For Oxford Nanopore sequencing, high-molecular-weight DNA was isolated using a BluePippin system (Sage Science, Beverly, MA, USA). Approximately 1.5 μg of genomic DNA was used for library construction using a one-dimensional (1D) ligation sequencing kit (SQK-LSK109; Oxford Nanopore Technologies [ONT]). No size selection or shearing was applied. The library was loaded into an R9.4 flow cell for the PromethION platform (PromethION flow cells, FLO-PRO002; Oxford Nanopore). After base calling by Albacore v4.3.2, a total of 25,086 reads with an average length of 39,864 bp and an *N*_50_ value of 39,304 was obtained (11). Nanopore quality control was achieved using NanoPlot v1.15.0 with a threshold value (Q) of >7 (12).

For Illumina sequencing, 1 μg DNA was used with the NEBNext Ultra DNA library prep kit (New England BioLabs) according to the manufacturer’s information. The Illumina library was sequenced on the Illumina NovaSeq 6000 platform at Benagen (Wuhan, China). Approximately 1.7 Gb of raw data of 150-bp-long paired-end reads were generated. A total of 5,521,000 reads were subjected to quality control and trimming using SOAPnuke 1.3.0 (13), which removed reads containing 50% low-quality bases (quality value, ≤5) and overlaps with adapter sequence, generating a total of 5,510,418 clean reads.

The assembly was completed with Unicycler 0.4.8 (14), yielding a single circular contig with a length of 3,364,212 bp and a GC content of 67.65%. The final coverage of the genome was 100%. The depth of Illumina sequence averaged 489.5×, while for Nanopore it averaged 282.42×. For all software used, default parameters were used except where otherwise noted. The annotation was performed with the NCBI Prokaryotic Genome Annotation Pipeline 4.12 (PGAP) (15), which predicted a total of 3,375 genes with 3,298 coding sequences and 49 RNA genes (3 rRNAs, 43 tRNAs, and 3 noncoding RNAs). Further sequence analysis revealed a photosynthesis gene cluster, including genes encoding bacteriochlorophyll a, suggesting *Sandaracinobacter* sp. strain M6 to be an aerobic anoxygenic phototroph.

### Data availability.

The complete genome sequence of *Sandaracinobacter* sp. M6 has been deposited at GenBank under accession number CP059851.1. The SRA deposit is available under accession numbers SRX9465014 and SRX9465015. The BioSample and BioProject accession numbers are SAMN15676387 and PRJNA649658, respectively.
